# Curcuminoids and ω-3 fatty acids with anti-oxidants potentiate cytotoxicity of natural killer cells against pancreatic ductal adenocarcinoma cells and inhibit interferon γ production

**DOI:** 10.3389/fphys.2015.00129

**Published:** 2015-05-22

**Authors:** Ramesh C. Halder, Anasheh Almasi, Bien Sagong, Jessica Leung, Anahid Jewett, Milan Fiala

**Affiliations:** ^1^Department of Surgery, University of California, Los Angeles, School of MedicineLos Angeles, CA, USA; ^2^Division of Oral Biology and Oral Medicine, The Jane and Jerry Weintraub Center for Reconstructive Biotechnology, School of Dentistry and Medicine, University of California, Los AngelesLos Angeles, CA, USA; ^3^The Jonsson Comprehensive Cancer Center, School of Dentistry and Medicine, University of California, Los AngelesLos Angeles, CA, USA

**Keywords:** pancreatic cancer, curcumin, ω-3 fatty acids, natural killer cells, interferon-γ

## Abstract

Pancreatic cancer has a poor prognosis attributed in part to immune suppression and deactivation of natural killer (NK) cells. Curcuminoids have a potential for improving the therapy of pancreatic cancer given promising results in cancer models and a clinical trial, but their oral absorption is limited. Our objective in this study is to show curcuminoid anti-oncogenic effects alone and together with human NK cells. We tested curcuminoids in an emulsion of ω-3 fatty acids and anti-oxidants (“Smartfish”) regarding their direct cytocidal effect and enhancement of the cytocidal activity of NK cells in pancreatic ductal adenocarcinoma (PDAC) cells (Mia Paca 2 and L3.6). Curcuminoids (at ≥10 μM) with ω-3 fatty acids and anti-oxidants or with the lipidic mediator resolvin D1 (RvD1) (26 nM) induced high caspase-3 activity in PDAC cells. Importantly, curcuminoids with ω-3 fatty acids and anti-oxidants or with RvD1 significantly potentiated NK cell cytocidal function and protected them against degradation. In a co-culture of cancer cells with NK cells, interferon-γ (IFN-γ) production by NK cells was not altered by ω-3 fatty acids with anti-oxidants or by RvD1 but was inhibited by curcuminoids. The inhibition was not eliminated by ω-3 fatty acids or RvD1 but was relieved by removing curcuminoids after adding NK cells. In conclusion, curcuminoids with ω-3 fatty acids and anti-oxidants or with RvD1 have increased cytotoxic activity on PDAC cells alone and with NK cells. The effects of curcuminoids with ω-3 fatty acids and anti-oxidants on pancreatic cancer will be investigated in a mouse model with humanized immune system.

## Introduction

Pancreatic ductal adenocarcinoma (PDAC) is the 4th leading cause of cancer-related deaths in the United States with an overall survival of less than 1 year and based on current statistics is predicted to become the 2nd leading cause of cancer-related deaths by 2020 (American Cancer Society, 2012). Chemotherapy has been less successful in PDAC in comparison to other cancers. The first line drug gemcitabine increased overall 1-year survival to 18% for gemcitabine-treated patients in comparison to 2% for 5-fluorouracil-treated patients (Burris et al., 1997).

The inflammatory milieu in pancreas may precede cancer onset due to chronic pancreatitis and be intimately involved with cancer progression. Although initially the immune cells tend to eliminate cancer cells, they ultimately fail and actually promote oncogenesis through IL-6 (Sideras et al., 2013). The immune system is modulated by cancer to inhibit the cytotoxic function of natural killer (NK) cells and CD8 T cells through activation of regulatory T cells, myeloid derived suppressor (MDSC) cells, tumor associated macrophages and fibroblasts. Indeed, the cytotoxicity of NK cells from tumors and peripheral blood of cancer patients is significantly reduced (Lai et al., 1996; Jewett et al., 2013). The immune therapies of pancreatic cancer using vaccination with cancer proteins and inactivated allogeneic cancer cells and adoptive cell therapy using cytotoxic T cells have not produced clear results in short-term trials (Sideras et al., 2013). Thus, new approaches to pancreatic cancer treatment are urgent.

Curcuminoids are under intensive study against cancer as they inhibit cell proliferation through suppression of NFκB and of a NFκB-dependent protein Akt, and suppress anti-apoptotic genes (Aggarwal et al., 2006). By activation of various transcription factors, curcuminoids target manifold proteins *in vivo* (Goel et al., 2008). In a rat model, curcuminoids attenuated the severity of pancreatitis (Gukovsky et al., 2003). Curcuminoids produced promising results in pancreatic cancer patients in combination with gemcitabine (Kanai et al., 2011). However, the plasma concentrations of curcuminoids achieved in that study were very low (<0.5 μg/ml). Thus, proper formulation of curcuminoids for increased absorption, stability and cellular activity is needed for clinical applications. In addition, curcuminoids block the production of the cytokine interferon-γ that has a key role in anti-oncogenic differentiation of cancer cells. Interferon-γ is a member of the type II interferon family with its own receptor, which signals through STAT-1 to transactivate IFN response factor 1 (IRF1) and downstream secondary response genes. (Pestka et al., 2004). Biological activities involve anti-proliferative, anti-angiogenic, and proapoptotic effects against cancer cells (Chawla-Sarkar et al., 2003). However, IFN-γ has also negative pro-tumorigenic effect in cancer (Maio et al., 1991; Zaidi and Merlino, 2011). Docosahexaenoic acid (DHA) and eicosapentaenoic acid (EPA) are ω-3 fatty acids with anti-cancer properties through production of toxic intermediates (Gleissman et al., 2010) and specialized proresolving mediators (SPMs) called resolvins, such as resolvin D1 (RvD1). Resolvins are lipidic mediators which resolve acute inflammation and may prevent chronic inflammation leading to cancer (Greene et al., 2011). The Western diet lacks DHA and EPA for production of SPMs. The low ratio of ω-3/ω-6 fatty acids in the Western diet promotes macrophage recruitment and activation of tumorigenic pathways. As changing the diet is difficult for many people, supplementation with ω-3 fatty acids may optimally increase the ω-3/ω-6 ratio in the diet.

Both curcumin and lipidic mediators from ω-3 block signaling by NFκB but their anti-oncogenic effects have not been compared. Here we have examined whether the combination of curcuminoids with ω-3 fatty acids and anti-oxidants will increase cytotoxicity directly and/or synergistically with NK cells.

## Materials and methods

### Cell culture

#### Pancreatic cancer

Pancreatic cancer cells called Mia Paca2 (MP2) and L3.6 PDAC cells were propagated in DMEM with 10% fetal calf serum and antibiotics.

### Human subjects

The human investigations were performed after approval by the UCLA institutional review board #11-000005 and in accordance with an assurance filed with and approved by the U.S. Department of Health and Human Services. Thirty healthy donors and a patient with disseminated prostate cancer were recruited into the study after signing an informed Consent.

### NK cells

NK cells were isolated from the blood of 30 healthy subjects by negative selection using the NK cell isolation kit (Stem cell technologies, Vancouver, Canada). NK cells were activated by treatment with IL-2 [termed NK(IL-2)] or IL-2 and CD16 antibody [termed NK(IL2CD16)] (BioLegend, San Diego, CA) as described (Tseng et al., 2010). NK (IL-2) cells are activated for cytotoxicity. NK (IL-2CD16) have decreased cytotoxicity but increased cytokine production (“split anergy”).

### Lipidic emulsions

Smartfish^R^ (Smartfish Company, Oslo, Norway), a lipidic emulsion with DHA (1 gm/200 ml) and EPA (1 gm/200 ml) (from local cod and salmon) protected against oxidation by botanical anti-oxidants (polyphenols, pectin and whey proteins, tocopherols and rosemary extract, pomegranate, chookberry) and extremely low in environmental toxins; Smartfish/CUR^R^ containing a supplement of curcuminoids (760 mg /200 ml); control fish-oil emulsion (from imported sardines and anchovies not protected against oxidation). For use in cell culture, each emulsion was diluted 1:2 with fetal calf serum, sonicated for 30 s and then diluted 1:100 in DMEM with 10% fetal calf serum. For nutritional supplementation, the prostate cancer patient was taking one carton of the drink Smartfish/CUR^R^ daily.

### Nutritional substances

Curcuminoids (prepared from Curcuma longa L root by extraction with acetone as granulated powder by Naturex, Avignone, France) were prepared by sonication as an emulsion in Smartfish^R^ by the Smartfish company. Resolvin D1 (RvD1) (Cayman Chemical company, Ann Arbor, MI) was emulsified in Smartfish^R^.

### Direct cancer cell cytocidal assay by curcuminoids and ω-3

MP2 cells (10,000 cells) were plated in each well of a 24-well plate and were grown at 37 C 24 h before testing. Triplicate MP2 cell cultures were treated for 24 or 48 h with curcuminoids, RvD1, or other additives, harvested into the lysis buffer and tested by the enzymatic caspase-3 assay.

### Combined cancer cell cytocidal assay by NK cells together with curcuminoids and ω-3

MP2 cell cultures were prepared as above. NK cells were added to MP2 cells at a ratio of 5 NK cell per 1 MP2 cancer cell. The co cultures were treated with various additives for 24 or 48 h and processed as in the direct assay.

#### Enzymatic caspase-3 assay

Enzymatic caspase-3 assay was performed by the CaspACE-3 assay G-7351 (Promega Corporation, Madison, WI) according to the manufacturer's instructions.

### Caspase-3 immunofluorescent assay

MP2 cells were grown to partial confluence in 8-well chamber slides (Corning) in DMEM with 10% fetal calf serum and were treated 24 h as stated. Then they were fixed by 4% paraformaldehyde, permeabilized using 0.25 Triton, stained using the indirect technique with rabbit anti-caspase-3 (active) (Genetex, Irvine, CA) at 1:200 dilutions followed by ALEXA-fluor-donkey anti-rabbit 568 (InVitrogen, Carlsbad, CA) at 1:200 dilutions and FITC-phalloidin (Sigma, St. Louis, M0) at 1:500 dilution, mounted using Prolong Gold antifade with DAPI (In Vitrogen, Carlsbad, CA). The slides were examined using Olympus B-max fluorescence microscope at 20× and 40× magnification with the Hamamatsu camera, and fluorescence was determined in four fields using Image Pro software and expressed as Integrated Optical Density (IOD) per macrophage.

### Interferon-γ assay

Supernatants were tested by interferon-γ ELISA assay (BioLegend, San Diego, CA).

### Statistical analysis

Means were compared using analysis of variance methods. Means and their approximate 95% confidence bounds (mean ± 2 standard errors of the mean) are reported. Computations were carried out using JMP software (SAS Inc, Cary, NC).

## Results

### Curcuminoids or RvD1 have increased cytocidal activity against pancreatic cancer cells when emulsified in ω-3 with anti-oxidants

**Figure 1:**
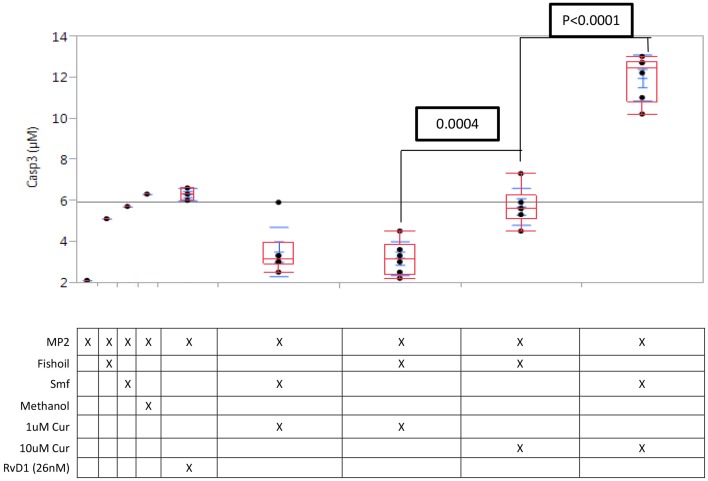
**Caspase-3 induction in MP2 cells is potentiated by curcuminoids with ω-3 and anti-oxidants**. Replicate MP2 cell cultures (*N* = 3) in 24-well plates were treated 24 h with curcuminoids in Smartfish emulsion or fish oil, the cells were harvested and caspase-3 was assayed in the whole lysate. The experiment was repeated twice with comparable results. The maximal induction of caspase-3 reached by 10 μM curcuminoids in Smartfish was greater than any other treatment (*P* < 0.0001).

We tested apoptosis induction using the enzyme assay for the executioner caspase-3 expression. In MP2 cells alone, caspase-3 expression was low (1.2 μM) and was not significantly increased by curcuminoids (1 μM) alone or with ω-3 and anti-oxidants, or by fish oil; methanol is used as a control However, curcuminoids (10 μM) emulsified in ω-3 and anti-oxidants induced robust capase-3 expression, which was significantly higher (*P* < 0.0001) than that induced by curcuminoids (10 μM) in fish oil (Figure 1).

**Figure 2:**
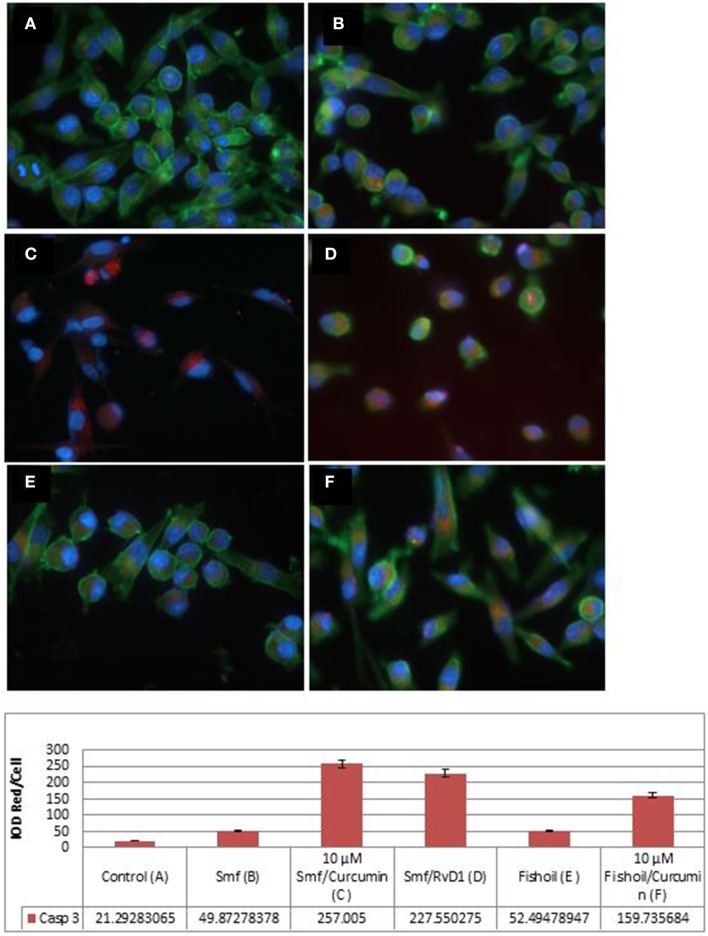
**Active caspase-3 expression in MP2 cancer cells is potentiated by curcuminoids and ω-3 or Resolvin D1**. The MP2 cells were stained by phalloidin (green) and by rabbit antibody to active caspase-3/anti-rabbit ALEXA568 (red). (**A)** Untreated MP2 cells; (**B)** MP2 cells treated by ω-3 with anti-oxidants (Smartfish); (**C)** MP2 cells treated by curcuminoids with ω-3 and anti-oxidants (Smartfish); (**D)** MP2 cells treated by RvD1 and ω-3 with anti-oxidants(Smartfish); (**E)** MP2 cells treated by fish-oil; (**F)** MP-2 cells treated by curcuminoids in fish oil. Note the highest expression of caspase-3 in **(C,D)**.

We examined apoptosis by immunofluorescence microscopy of caspase-3 antibody stained cells (Figure 2). As shown in integrated optical density (IOD) of red per cell, caspase-3 expression was not observed in untreated MP2 cells (IOD Red/Cell = 21.29), but was seen at a low level in MP2 cells treated with ω-3 and anti-oxidants (IOD Red/Cell = 49.87), and at a high level in MP-2 cells treated with curcuminoids or RvD1 emulsified in ω-3 with anti-oxidants (IOD Red/Cell = 257.01 and 227.55, respectively). The cancer cells treated by ω-3 and anti-oxidants became rounded, detached and some displayed pyknotic nuclei confirming their apoptosis. The emulsification of curcuminoids in fish oil was clearly inferior in the production of apoptosis (IOD Red/Cell = 159.74). Comparable results were obtained in three experiments.

### Curcuminoids with NK cells are more cytocidal in cancer cells than curcuminoids or NK cells alone

**Figure 3:**
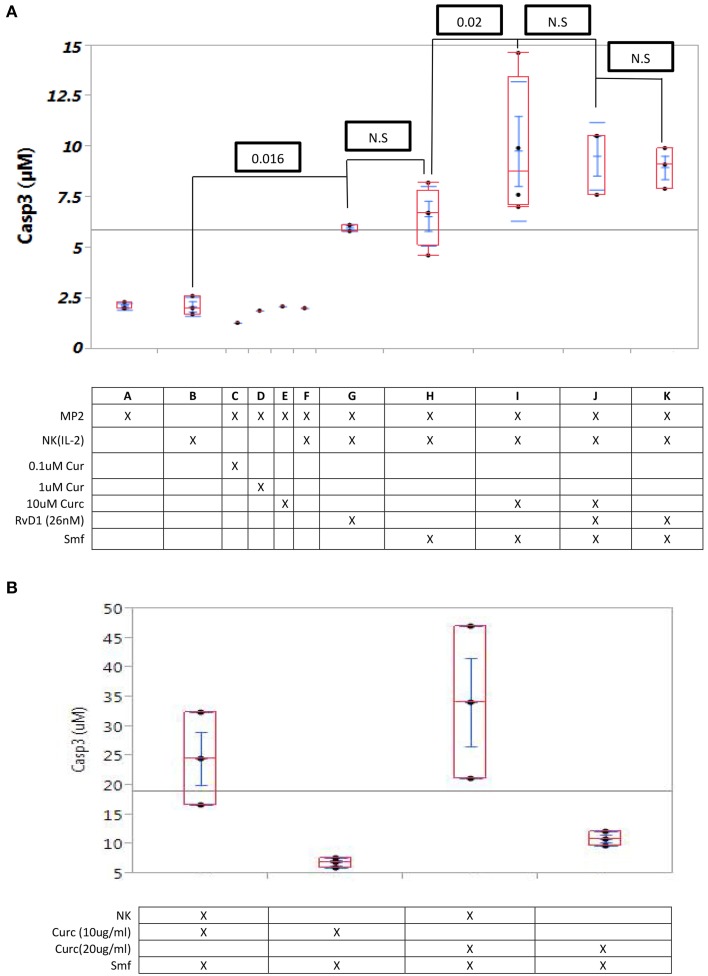
**Curcuminoids or RvD1 potentiate caspase-3 induction in MP2 cells by NK cells. (A)** Caspase -3 induction in MP2 cells: Quadruplicate MP2 cell cultures in 24-well plates were co cultured 24 h with NK cells (5 NK cells/1 cancer cell) with ω-3 emulsion and anti-oxidants and with or without curcuminoids or RvD1. (A–F) No caspase-3 induction by NK cells alone or curcuminoids alone; (G,H) low induction by NK cells with ω-3 or RvD1 (26 nM) (*P* = 0.016); (I–K) maximum induction by NK cells with ω-3or RvD1 (*P* = 0.02). (**B)** Comparison of caspase-3 induction by curcuminoids and smartfish with or without NK cells.

In MP2 cells alone, MP2cells with NK (IL-2) cells, and MP2 cells with curcuminoids (0.1–10 μM), caspase-3 expression was low (means 2.0–3.1 μM) (cf. A through F in Figure 3A). Caspase-3 expression was higher in the co culture of MP2 cells with NK (IL-2) cells in ω-3 and anti-oxidants or with RvD1 (means 6.0–6.6 μM) (cf. G and H in Figure 3A) and in a co culture of MP2 cells with NK (IL-2) cells and curcuminoids. However, the cytocidal activity of NK(IL-2) cells was significantly increased by curcuminoids (*P* = 0.02) in ω-3 and anti-oxidants (mean = 9.8 μM), in ω-3, anti-oxidants, and RvD1 (mean = 9.0 μM) or in ω-3 and RvD1 (mean = 9.5 μM) (cf. I through K in Figure 3A). Comparable results were obtained in four experiments with NK cells, curcuminoids, ω-3 and anti-oxidants.

A comparative study of cytotoxicity by curcuminoids alone (in ω-3 and anti-oxidants) vs. curcuminoids (in ω-3 and anti-oxidants) and NK cells demonstrated that curcuminoid-enhanced NK cell cytotoxicity was greater than direct curcuminoid cytotoxicity (Figure 3B). The experiment was repeated three times with comparable results.

### ω-3 fatty acids with anti-oxidants protect NK cells and increase apoptosis of cancer cells

**Figure 4:**
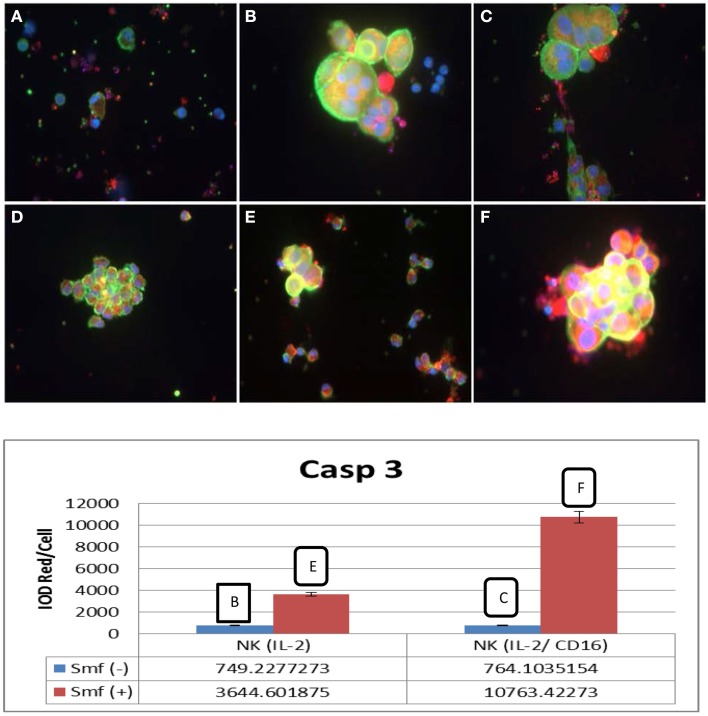
**Induction of active caspase-3 in L3.6 cancer cells by NK cells and potentiation by ω-3 and anti-oxidants. (A)** NK(IL-2) cells alone; **(B)** NK(IL-2) cells and L3.6 cells; **(C)** NK (IL-2/CD16) cells and L3.6 cells, **(D)** NK cells with ω-3 and anti-oxidants, **(E)** NK(IL-2) cells and L3.6 cells with ω-3 and anti-oxidants; **(F)** NK(IL-16) and L3.6 cells with ω-3 and anti-oxidants. Phalloidin (green) and caspase-3 (red). NK cells are small green cells; cancer cells are large green cells. Caspase-3 is yellow or red. Red pixels were scanned by Image-Pro.

We examined caspase-3 induction by immunofluorescence microscopy. Small NK cells are distinguished by immunofluorescence microscopy from large L3.6 cancer cells. Both cells are stained green in the cytoplasm and blue in the nuclei. In this experiment (Figure 4), NK cells were incubated overnight in DMEM with ω-3 and anti-oxidants (Smartfish) either alone or with cancer cells. NK cells alone remained intact when incubated in DMEM with ω-3 and anti-oxidants in comparison to NK cells incubated in DMEM (cf. A–D and B–E in Figure 4). In a co culture of NK(IL-2) or NK(IL-2CD16) cells with L3.6 pancreatic cancer, cancer cells showed increased caspase-3 expression. Active caspase-3 was shown by increased red or yellow color in the cytoplasm of cancer cells stained with caspase-3 antibody (cf. B–E and C–F in Figure 4) when incubated in the presence of ω-3 and anti-oxidants in comparison to medium as shown in integrated optical density (IOD) of red per cell. In case of NK(IL-2), IOD increased from 749.23 to 3644.60 in the presence of ω-3 and anti-oxidants, and in case of NK(IL-2/CD16), IOD increased from 764.10 to 10763.42 in the presence of ω-3 and anti-oxidants (Figure 4).

### Curcuminoids inhibit the production of interferon-γ by NK cells during co-incubation with MP2 cancer cells

**Figure 5:**
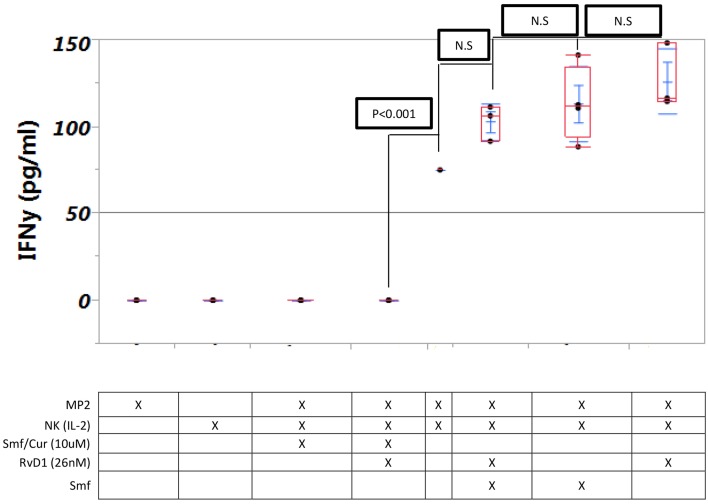
**Interferon-γ production by NK cells is not changed by ω-3 or RvD1 but is blocked by curcuminoids**. Triplicate MP2 cell cultures in 24-well plates were cultured with NK cells with or without Smartfish emulsion, curcumin, or RvD. Note blockade of IFN-γ by curcumin.

No IFN-γ was produced by NK (IL-2) cells alone. High level of IFN-γ was produced by NK(IL-2) cells in a co culture with cancer cells with or without ω-3 and anti-oxidants or RvD1. Curcuminoids (10 μM) with or without ω-3 and anti-oxidants or RvD1 completely blocked production of IFN-γ (Figure 5).

**Figure 6:**
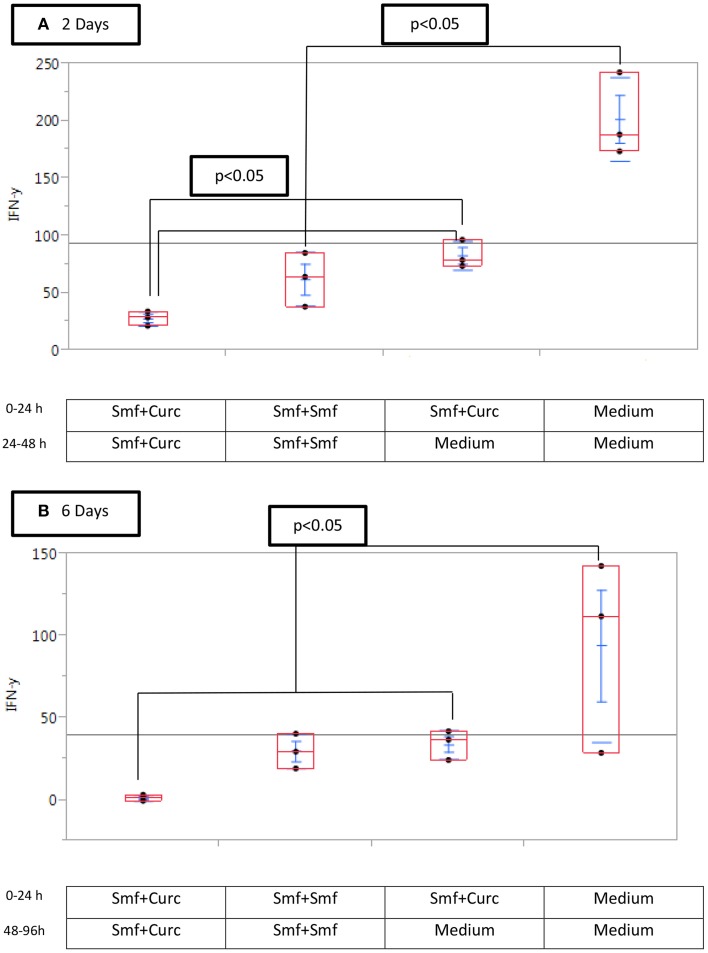
**Curcuminoid blockade of interferon-γ production is reduced by removal of curcumin (A) after 2 days, (B) after 6 days**. Triplicate MP2 cell cultures in 24-well plates were cultured with or without Smartfish emulsion and curcuminoids or with DMEM, washed and replaced with the indicated medium and NK cells for 24 or 48 h. Note that the inhibition of IFN-γ by curcuminoids was reduced by washing MP2 cells.

We tested the effect on IFN-γ production of incubation of the cells in a curcuminoid-free medium after addition of NK cells. The curcuminoid-free interval after addition of NK cells resulted in the following mean IFN-γ levels: (a) co culture of NK and cancer cells without curcuminoids for 48 h: 201 pg/ml; (b) co culture without curcuminoids for 24 h: 90 pg/ml; (c) co culture with constant presence of curcuminoids for 48 h: 27 pg/ml (Figure 6A). When the experiment was increased to 96 h, the reduction in inhibition was still observed (Figure 6B).

## Discussion

We evaluated the induction of the apoptosis executioner caspase-3 by curcuminoids and ω-3 or the lipid mediator RvD1 in PDAC cells as (a) direct effects, and (b) combined effects with NK cells. The analysis of direct effects showed that curcuminoids alone were poorly effective but had strong cytotoxic effects in dose-related fashion when administered in an emulsion of ω-3 with anti-oxidants in comparison to an emulsion in fish oil. The cytotoxic activity of NK cells was enhanced by ω-3 and anti-oxidants and was maximally enhanced by curcuminoids and ω-3 fatty acids with anti-oxidants. Thus, NK cells either with curcuminoids, ω-3 fatty acids and anti-oxidants or RvD1 (at 1000 lower concentration) induced maximal caspase-3. We also evaluated cytotoxicity by immunofluorescence microscopy in MP2 and L3.6 cancer cells. The results confirmed the increase of cidal effects from the culture in ω-3 and anti-oxidants to the culture in ω-3, anti-oxidants and curcuminoids (Figure 2) and from the culture with NK(IL-2) cells to NK(IL-2)cells, ω-3, anti-oxidants (Figure 4). Caspase-3 was activated in both cancer cells and NK cells, but the activation was much increased by ω-3 and anti-oxidants or RvD1 (Figure 3A). In addition, NK cells were protected against degradation after overnight incubation in DMEM with ω-3 and anti-oxidants in comparison to DMEM (Figure 4). Although previous studies showed potentiation of cytotoxic effects of NK cells against cancer cells by curcuminoids (Zhang et al., 2007), in our study these effects were further enhanced by the synergy between curcuminoids and ω-3 or RvD1. A previous study demonstrated an increase by curcumin of the caspase-8, which is upstream of caspase-3 in the execution-phase of apoptosis (Kim et al., 2005).

Curcuminoids inhibited the production of IFN-γ by NK cells in our study of pancreatic cancer cells and in a study of human melanoma cells (Bill et al., 2009). The latter group prepared a curcumin analog FLLL32 which does not block IFN-γ production by NK cells (Bill et al., 2010). The effect of curcuminoids on IFN-γ was reduced when curcuminoid treatment was reduced in time after the addition of NK cells (Figure 6).

In conclusion, our cell culture study showed dissociation between enhancing effects of curcuminoids on apoptosis and inhibiting effects on IFN-γ production by NK cells. In application to cancer therapy, it will be important to determine whether the inhibitory effect of curcuminoids on IFN-γ production could be modified by intermittent therapy. In the future work, we plan to study the cytocidal effects of curcuminoids, ω-3, and anti-oxidants *in vivo* in an animal model and ultimately in human patients. We are preparing such study in a model of pancreatic cancer with humanized immune system.

### Conflict of interest statement

The authors declare that the research was conducted in the absence of any commercial or financial relationships that could be construed as a potential conflict of interest.
